# Social media interactions between government and the public: A Chinese case study of government WeChat official accounts on information related to COVID-19

**DOI:** 10.3389/fpsyg.2022.955376

**Published:** 2022-09-06

**Authors:** Chang’an Shao, Xin Guan, Jiajing Sun, Michael Cole, Guiying Liu

**Affiliations:** ^1^School of Economics Management, Institute of Disaster Prevention, Sanhe, China; ^2^School of Public Policy and Management, University of Chinese Academy of Sciences, Beijing, China; ^3^School of Economics and Management, University of Chinese Academy of Sciences, Beijing, China; ^4^Management School, University of Liverpool, Liverpool, United Kingdom

**Keywords:** public energy field, COVID-19, social media, social discourse system, WeChat official account, text analysis

## Abstract

The concept of a *public energy field* is central to public administration discourse theory. Its main idea is the facilitation of dialog between government and the public, on the basis of equality, to construct a public policy consensus. In contemporary society, social media provides new and distinctive channels for such interactions. Social media can, therefore, be conceived as a novel type of *public energy field*. Since the outbreak of the COVID-19 pandemic, interactions between the Chinese government and the Chinese public (whether located in China or abroad) have been acutely reflected through WeChat official accounts. This article focuses on the COVID-19 pandemic and, through social media text mining and processing, analyzes the text topics and emotions covered. Basic principles of discourse validity, regarding this public energy field and two guarantee conditions, are applied to analyze the information exchange and dialog between the government and the public on COVID-19 through WeChat official accounts. It was found that the government’s WeChat official accounts have not yet formed a harmonious dialog space to balance the public energy field, and that the interaction between the government and the public has to be improved. The government’s social discourse had a significant influence on the public’s social discourse. Using text analysis, the study found that the government has published information on 11 topics related to the prevention and control of the pandemic. It can be argued that the public energy *field* presented by both the government and the public effectively portrayed and reflected the actual situation of the pandemic in China.

## Introduction

Since the outbreak of COVID-19, the Chinese government has taken a series of measures to combat the pandemic. The rapid development of information technology has increased the complexity of social and political discourse, and given the government unprecedented challenges. This crisis posed risks for the government that public trust might erode if confidence in the handling of the pandemic, and or faith in the accuracy and/or timeliness of official information, declined. One strategy to forestall such an outcome was to involve the public more intensively in the policy debate (Halvorsen, 2003). Such an enhancement of public participation (Barnes et al., 2003) has also been widely recognized as a route to facilitate genuine improvements in policy outcomes and can be interpreted as a method to improve the efficiency of government and the quality of public services (Nayak and Samanta, 2014; Hue and Sun, 2022). Interaction between government and the public is increasingly conducted through social media; a trend that was acute concerning the COVID-19 pandemic, given the social-distancing restrictions.

Social media has been playing an increasingly significant role in our modern society (Yang et al., 2020). “Sina Weibo” and “WeChat” are two of the most popular and important social media platforms in China (Carvajal-Miranda et al., 2020). Some of their features and services overlap (Gan, 2018), for instance, functions including chatting, sharing, commenting, and so on. Many government departments have been using Sina Weibo or WeChat to interact with the public and achieving remarkable results.

However, WeChat, regarded as an efficient instant messaging tool, also can be used to publish information and provide services. Moreover, more than 1.2 billion users from WeChat ensures that information is widely disseminated (Jiang et al., 2021); hence it increases the diversity of the user groups, the richness of user groups, and the engagement of the users. In addition, Sina Weibo is more likely to be threatened by a discourse monopoly, while the discourse center of WeChat is more multidimensional (Ma, 2014).

Nowadays, the public and institutions are more inclined to use WeChat official accounts or WeChat applets. For instance, Zhang et al. (2018) demonstrated that hospitals are more likely to use WeChat than Weibo. During the pandemic, the Health QR Code and Travel Card, which were widely used, were all based on the WeChat platform. Therefore, the article selected WeChat as the research object.

In China, WeChat official accounts form a vital interactive platform for the government and its agencies to disseminate information on time, and for the public to engage in political and social debates. Such interactions form the *public energy field* where public policies can be formulated and modified through social discourse, which, at least occasionally, challenges governmental agendas and practices (Fox and Miller, 1994).

This article contributes to our understanding of interactions between government and the public during the COVID-19 pandemic in China. During the severe period of the pandemic, information related to the emergency released by the government and media was eagerly received by the public and there was a widespread desire to engage in debates about the pandemic (Lu and Zhang, 2020). Most of this informational exchange occurred *via* WeChat official accounts. We deploy four principles and two guarantee conditions to study the *public energy field* contained within the social media discourse. Discourse analysis is undertaken on the interactive information flow between the government and the public, through WeChat official accounts, for the COVID-19 pandemic. We consider both governmental and public perspectives to evaluate this discourse.

## Literature review

### Discourse systems in social media

The concept of social discourse reflects oral or written communication that has a social purpose or a distinctive social component (Addams and Proops, 2000). With the rapid development of the Internet, social media has been increasingly adopted as a core mechanism for social discourse on central aspects of contemporary political and public policy debate. Social media provides channels for information release and social interchanges and establishes a platform for political discourse, thereby facilitating the reconstruction of social/political relations, blurring the boundaries between the real and the virtual (Trilling, 2014), and creating their *public energy field.*

The Internet offers the potential of an approximately equal space for individuals and organizations. Social media creates new challenges and opportunities for public administrators (Knox, 2016), such as the enhancement of policy information (Gong et al., 2022), and increases the capacity of the public to participate in public debates (Mossberger et al., 2013). People’s active participation establishes a unique political/social discourse system in cyberspace covering a variety of media formats (including text, audio, image, and video). By mining the discourse within these media, the contribution of this communication to public debates can be identified, the importance of such forums highlighted, and the capacity to improve government policies specified (Kavanaugh et al., 2012). Such methodologies have also enabled scholars to develop overarching conclusions about the impact of social media, for instance, Li et al. (2019) observed that interactions between the public and governments in China reflected specific and changing social–political discourse and political values. In terms of three separate self-identities – individualism, relational collectivism, and group collectivism – expressed through such discourses, those authors found that the Chinese government was more inclined to respond to demands articulated through relational collectivism. That is, the government was most responsive to agendas emerging from networks constructed through interpersonal relationships, rather than a wider sense of belonging to a collective entity (Kreuzbauer et al., 2009). Dialog and discourse in social media can, thus, have a positive influence on promoting interaction between the public and government, and improving relationships between them. Such an outcome is normally specified as a core goal of digital governance (Chadwick, 2009).

Nevertheless, encouraging the public to actively participate in such social media interaction remains a core challenge for policy-makers (Mossberger et al., 2013). Perhaps the most effective strategy has involved government extensively promoting dialog on issues that were of immediate relevance to a wide social segment, rather than broader organizational interests or marketing-related matters (Bonsón et al., 2015).

### Public energy field

The idea of a *public energy field* is central to discourse theory (Fox and Miller, 1994), which represents an interdisciplinary application of postmodern discourse to contemporary international society. *A public energy field* is a political term formed by the combination of the field theory of modern physics and phenomenological methodology. Its purpose lies in the creation of a distinctive discourse system, which emphasizes the public voice and advocates independent expression. *Public* is a field composed of action and discourse, which confronts elite authority and is open to all citizens. *Energy* refers to the internal force and the collision force, while *Field* concerns the synthesis of forces acting on the situation. The structure of a field is not a fixed formula but depends on what is happening (Fox and Miller, 1994).

### Discourse legitimacy

The access mechanisms of discourse platforms provided by social media are open and free. However, scholars such as Fox and Miller (1994) suggested, constructed through Habermas’ specification of valid conditions for ideal speech and authentic communication, four practical claims (warrants) for valid discourse – sincerity; situation-regarding intentionality; willing attention; and substantive contribution. Sincere discourse required mutual trust to safeguard the public interest, which is undermined through the proliferation of disingenuous arguments (Fox and Miller, 1993). Situation-regarding intentionality reflects requirements that the dialog concerns an issue that is “contextually situated” and that “speakers will take into account the context of the problem, the lives of those affected, and the public interest” (Fox and Miller, 1994, p. 123). In other words, those deliberations need to be grounded in the real world context rather than have an abstraction from reality. Requirements for willing attention relate to aspirations for “a spirit of vigorous, active even passionate engagement” (Fox and Miller, 1994, p. 125). Finally, the substantive contribution warrant recognises the requirements for informed dialog, for instance, through “offering a unique viewpoint, specific expertise, generalized knowledge, or pertinent life experience, or by being able to express the concerns of groups or classes or citizens” (Fox and Miller, 1994, p. 125). To secure the legitimacy of the discourse, Fox and Miller (1994) also specified two guarantee conditions. First, equality between the participants and, second, the dialog culture term *some people*, which reflected inclinations to avoid dialog restricted to elite participation or a dialog overwhelmed through a multiplicity of voices. One central aim was the avoidance of monologues and the facilitation of meaningful arguments and refutation. Public administration discourse should also avoid the one-way interaction between the administrators and the public.

*Public energy fields* can be contrasted with discourse structures prevalent within organizations and policy networks. Organizations have formal relational structures, while policy networks normally exhibit relatively stable relationships (Kickert et al., 1997; Sørensen and Torfing, 2011). *Public energy fields* also have a much greater level of openness of access than organizations or policy networks (Schaap, 2007). A diverse range of protagonists with contrasting agendas, intentions, and perspectives thus clash, debate, and argue, often with a repetitious favor as ingrained perspectives and interests are regurgitated. This active dialog between governance and the public thus represents a departure from the official monologues (Farmer, 1995) and restricted enthusiasm for public participation (Cole, 2004) that has traditionally characterized bureaucratic behavior.

### Public crisis events and social media

For major public crisis events, the government must efficiently inform the public about the crisis (Chen et al., 2020). Social or electronic media often plays a crucial role in the rapid dissemination of information. For example, during the Haiti earthquake in January 2010, CNN’s website saw a significant increase in page views (about 240%) in 1 day (Bunz, 2010). When public crisis events occur, discourse in such media can reflect official attitudes and public feedback quickly, which assists in the dissemination of information to the public and the government, in terms of shifting public attitudes. Social media text analysis can be utilized to establish a link between informatics and disaster management (Gründer-Fahrer et al., 2018). Text analysis in social media brings new opportunities for crisis management. For instance, Laudy et al. (2017) introduced a series of text analysis tools, such as the tweet locator, to support the management of social media in a public crisis. These tools might be deployed to classify and manage the text, thus avoiding information overload as a consequence of a deluge of data.

Many scholars have used text analysis to study crisis events. For example, Mollema et al. (2015) studied relationships between the amount of social media information and measles reports during the measles outbreak among orthodox Protestants in the Netherlands, finding that data extracted from social media facilitated understanding of public attitudes. This information enabled public health institutions to respond to public concerns immediately. Similarly, Gründer-Fahrer et al. (2018) used natural language processing to mine relevant texts, and extract topics from Facebook, during the 2013 floods in China and Europe, thereby specifying the public’s emotional and wider response to that natural disaster and official measures. In the case of the Malaysian Airlines MH17 disaster, Jong et al. (2016) evaluated the response of local authorities in the Netherlands through social media and newspaper articles, finding that mayors could use social media to achieve crisis communication goals and operate as chief mourners for their communities.

Some studies have explored the weakness of government in social media interactions. Lachlan et al. (2016) discussed the use and content of Twitter in the pre-crisis stage of Storm Nemo, which hit the North Eastern United States and the Atlantic coast of Canada in 2013, finding that social media provided an opportunity to inform and motivate the public in the crisis events. Furthermore, those scholars asserted that emergency management officials should continue to search for specific and more general social media audiences, and consider the search strategies that affected audiences might deploy to facilitate information dissemination. Similarly, Chen (2012), through the analysis of social media coverage of the *Yongwen* railway accident in Wenzhou, China, concluded that social media played a crucial role in crisis communication. However, governmental inexperience with the technology meant that its communication appeared passive and lacking in adequate preparation.

## Conceptual framework

WeChat official accounts enable a dialog between followers and government-sponsored accounts through *comments* and facilitate the expression of emotions through *thumb-ups*. Texts published or the information released by the WeChat official accounts can be shared with others by the readers/followers. These interactive processes constitute social discourse, while the sheer volume and complex dynamics of these social discourses imply an interpretation as a *public energy field*.

Taking the COVID-19 pandemic as an example, the government supplied relevant information through the official account platforms. The government sought, therefore, to satisfy public demands for information about the pandemic and diminish unease about how the officials were handling the crisis. Furthermore, it might be argued that the dialog between the government and the public followed the principles of sincere interaction, the situation intentionality, willing attention and substantive contribution. Sincere interaction might be assumed given the widespread trust in the government among Chinese public opinion (Zhai, 2016), while the other principles might be credible assumptions given the immediacy of the emergency, the participatory enthusiasm, and the scale of the interaction.

**FIGURE 1:**
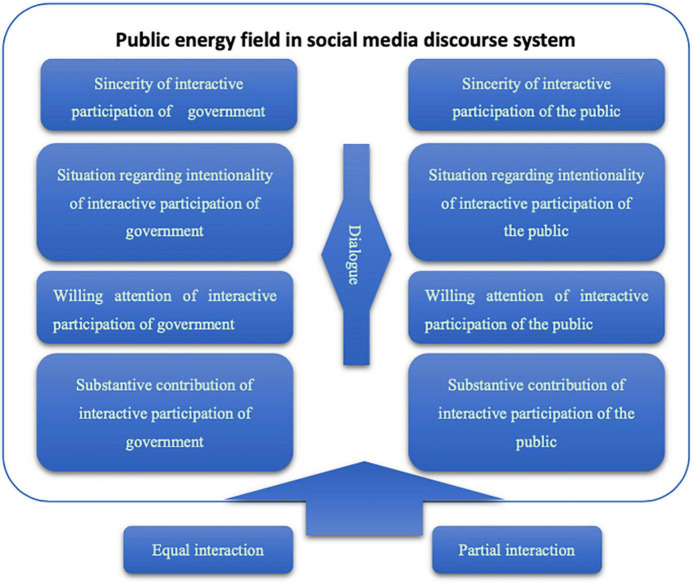
Research framework.

To evaluate these debates about COVID-19, we thus designed a *public energy field* with proxies for the four principles and guarantee conditions of discourse legitimacy (see Figure 1 and Table 1).

**TABLE 1:** Measurement index design of discourse legitimacy.

Practical claims for valid discourse (Fox and Miller, 1994)	Proposed framework (By authors)		
(a) sincerity		**Measurement index**	
(b) situation-regarding intentionality	**Elements**	**Government**	**The public**
(c) willing attention	(a) Sincerity of interactive participation	Associated with COVID-19 Position of text Guide to reading the full text	Active followers
(d) substantive contribution	(b) Situation regarding intentionality of interactive participation	Original content Number of videos Additional audio	Number of readings
	(c) Willing attention of interactive participation	Average daily number of texts Number of words in the text	Number of thumb ups Number of comments Number of thumb up comments
	(d) Substantive contribution of interactive participation	Importance of text Topic category of text Emotion of text	Number of lookings
The conditions for legitimate discourse (Fox and Miller, 1994)	(e) Equal interaction	Number of comment replies	Number of thumb up comment replies
(f) equality of discourse (g) some people’s discourse	(h) Partial interaction	Government response rate = Number of comment replies/Number of comments	Participation rate of the public = Number of comments/Number of readings

For the government, “Associated with COVID-19,” “Position of text” and “Guide to Reading the full text” were selected to indicate governmental sincerity in releasing information related to the pandemic. While for the public, the “Active followers” indicator was selected to specify the public’s sincerity in engaging those government accounts and, by implication, trust in the credibility of the government’s information on COVID-19 more generally.

For situation of interactive participation (or situation regarding intentionality), our government proxies were “Original content,” “Number of videos,” and “Additional audio,” to reflect government editing and “improving” the text quality. For the public, “Number of readings” was selected to indicate public participatory engagement with material directly affecting their lives.

In terms of participatory willingness (or willing attention), for the government “Average daily number of texts” and “Number of words in the text” were selected to describe texts and contents released by the government autonomously. Alternatively, for the public, this principle was proxied through “Number of thumb ups,” “Number of comments,” and “Number of thumbs up comments.” Thumb up behavior indicating active participation following receipt of the information.

To measure substantive contribution, “Importance of text,” “Topic category fo text,” and “Emotion of text” were chosen to describe how the government released information of varying importance, categories and emotions, and guides the public to read the contents. For the public, the “Number of lookings” indicator was selected as indicative of the scale of their engagement.

To evaluate the equality of the social media dialogue, “Number of comment replies” was selected to proxy official attitudes towards the status of public engagement, i.e. the seriousness or equality with which they interpreted public reactions. For the public, “Number of thumbs up comment replies” was selected to indicate to what extent the public sought to participate in a roughly equal dialogue with official sources.

Partial interaction, the *some people* idea espoused by Fox and Miller (1994), was proxied through “Government response rate” (Number of comment replies/ Number of comments), which identified willingness of the government to respond to public comments. In contrats, public participation was proxied through the Number of comments/the Number of readings.

## Public crisis events selection, research methods, and data

### Event selection and data collection

To evaluate social media communication surrounding the COVID-19 pandemic in China, and with reference to the influence ranking list of the government official account of “Xinbang” (^1^China’s most authoritative content industry service platform), we selected 10 influential government WeChat official accounts. Those sources were the China Government Network, the Communist Party Member, the Communist Youth League Central Committee, Beijing Release, Shanghai Release, Guangdong Release, Hubei Release, Wuhan Release, the Beijing Center for Disease Control and Prevention, and the Hubei Center for Disease Control. A total of 13,723 items of data for 114 days (from 8 January 2020 to 30 April 2020) were collected, incorporating basic data information, text information for the title and body, and information about government–public interactivity in relation to the text. Among the data collected, 3,988 items of information were not directly related to COVID-19, but mainly related to local economies and were largely collected in April, when the pandemic had been brought under control. Since the article aimed to describe the whole interaction situation between the government and the public during this period, all this data was analysed.

The timeframe reflected that, on 8 January 2020, the National Health Commission confirmed the new coronavirus as the source of the pandemic. On 20 January, Zhong Nanshan, the leader of the high-level expert team at the National Health Commission, clarified that the spread occurred through human-to-human transmission. After late January 2020, COVID-19 thus became a matter of acute public and governmental concern. However, after 30 April 2020 official data indicated that, in China, the pandemic was under control with few new infections and industry operating again. This period was, therefore, the critical phase in China’s domestic fight against the pandemic, and it was also the timeframe within which government, media, and public opinion were most concerned about the outbreak in China.

When the outbreak in China became relatively severe, the government and the media issued timely reports on pandemic-related information. The public exhibited acute concern about this information, and thus intensive willingness to participate in online interaction. Government official online social media, thus, provided rich research data and observation samples for studying the discourse system. Basic information about the 10 government WeChat official accounts is outlined in Table 2.

**TABLE 2:** Basic information of WeChat official account.

**Name of official account**	**Wechat certification authority**	**Estimated number of active followers** (Data from “Xinbang” and “Xi Gua Data”)	**Number of texts/Average daily number of texts** (The reference time period is 08 January 2020 to 30 April 2020)

Estimated number of active followers (Data from “Xinbang” and “Xi Gua Data”)	Number of texts/Average daily number of texts (The reference time period is 08 January 2020 to 30 April 2020)
China Government Network	China Government Network Operations Center of the General Office of the State Council	1691950	1137/10
Communist Party Member	Organization Department of the CPC Central Committee’s Party Member Education and Cadre Evaluation Center	1038150	952/8
Communist Youth League Central Committee	Central Committee of the Communist Youth League of China	3028900	1557/14
Beijing Release	The Information office of the Beijing Municipal Government	124183	1303/11
Shanghai Release	The Information office of the Shanghai Municipal Government	1244750	2499/22
Guangdong Release	The Information office of the Guangdong Municipal Government	3028900	945/8
Hubei Release	The Information office of the Hubei Municipal Government	162367	2287/20
Wuhan Release	The Information office of the Wuhan Municipal Government	110302	2334/20
Beijing Center for Disease Control and Prevention	Beijing Center for Disease Control and Prevention	113545	468/4
Hubei Center for Disease Control and Prevention	Hubei Center for Disease Control and Prevention	84042	241/2

The basic data items obtained through research and capture, calculation analysis, and manual reading judgments were classified as G (interactive behavior by the government) or P (interactive behavior by the public). The specific government categorization was as follows G1, Associated with COVID-19; G2, Position of text; G3, Guide to reading the full text; G4, Original content; G5, Number of videos; G6, Additional audio; G7, Average daily number of texts; G8, Number of words in the text; G9, Importance of text; G10, Emotion of text, G11, Number of comment replies; and G12, Government response rate. Similarly, for the public the categories were, P1, Active followers; P2, Number of readings; P3, Number of thumb-ups; P4, Number of comments; P5, Number of thumb-up comments; P6, Number of lookings; P7, Number of thumb-up comment replies; and P8, Participation rate of the public.

G1 and G4 were evaluated through manual reading (three people made initial judgments; after data correction, the data was finally determined, and a 100% consistency pass rate was obtained). G7, G12, and P8 were calculated and analyzed to obtain the final data. G9 and G10 were the final data of the text analysis of the title and body using Python. The remaining items were derived from the webpage data of the official accounts. G2 assigned data according to the importance of the text’s publication position (the headline is the most important, and so on). Finally, we studied the dimensionless processing of the data, so that the data mapping interval of all items is [0–100].

### Text processing and analysis

Next, we outline the Python processes for textual analysis.

(i) Word segmentation and cleaning:

We deployed the *jieba word segmentation database* to perform word segmentation; using Chinese stop vocabulary, Harbin Institute of Technology stop vocabulary, Baidu stop vocabulary, and Sichuan University Machine Intelligence Laboratory stop vocabulary. The aim was to clean the word segmentation results.

(ii) Text content analysis:

(1) The title:

We identified the 100 highest frequency words in title texts and assigned a weight to each of those words. Each high-frequency word had an average weight.

The importance of *Text*_*i*_ is calculated as follows:


$$TitleImportance_{i}=\sum_{w}w\times Weight_{w}\times TimesInTitle_{w}$$

where *i* refers to the *i*-th text, *w* stands for the *TimesInTitle*_*w*_-th high-frequency words, *Weight*_*w*_ represents the weight of *w*, and all weights are taken as 1, and *TimesInTitle*_*w*_ denotes the appearance of *w* in the title text frequency.

(II) Title and body:

We identified the 100 highest frequency words appearing in titles and the main body of the analysis, and assigned a weight to each high-frequency word (see Table 3).

**TABLE 3:** The first 100 high-frequency words statistics.

Word	Frequency	Word	Frequency	Word	Frequency	Word	Frequency
pandemic	75240	Related	11897	Discharged	8263	Policy	6493
Prevention and control	51411	Development	11840	People	8138	Area	6474
Job	40954	Country	11740	Support	8115	Center	6458
Wuhan	27276	Shanghai	11647	Construction	8108	WeChat	6450
pneumonia	26820	New	11418	Reporter	8080	Influence	6368
enterprise	24929	Infection	11149	Disinfect	8031	Way	6362
Case	24082	Measure	11001	Platform	7879	Ensure	6350
Hospital	23908	Beijing	10674	Unit	7831	Severe	6283
Personnel	20574	Source	10644	Grand total	7765	News	6150
COVID-19	18025	Coronavirus	10407	Department	7547	City	6124
Service	17409	Management	10370	Report	7512	Abroad	6046
Patient	17255	Add	10080	Produce	7374	Resume production	5999
China	16171	Information	9771	Treat	7274	Student	5958
Health	15990	Supply	9645	Treatment	7241	Meeting	5949
Confirmed	15717	Provide	9587	First line	7166	Advance	5940
Community	15662	Release	9566	Hubei province	7105	Fever	5906
Do well	14118	Period	9512	Activity	6952	Risk	5825
Time	14009	Organization	9467	pandemic prevention	6916	Community	5686
Situation	13692	Medical treatment	8983	Virus	6856	The masses	5648
Hubei	13629	Nationwide	8879	Protection	6794	School	5628
Mask	12617	Detect	8850	Xi Jinping	6727	Youth	5533
Isolation	12583	Implement	8670	Society	6710	Find	5454
Wuhan city	12230	Focus	8559	Citizen	6600	Further	5450
Guarantee	12214	Edit	8322	Enter	6545	Residents	5413
Resume work	12109	Life	8321	Hygiene	6541	Introduction	5316

The title and body text importance analysis data was assigned to the variable G9, Importance of text.

The importance of *Text*_*i*_ is calculated as follows:


$$G9_{i}=TextImportance_{i}$$

$$=\sum_{w}w\times Weight_{w}\times TimesInTitle_{w}$$

$$+\sum_{w}w\times Weight_{w}\times TimesInContent_{w}$$

where *i* represents the *i*-th text and *w* refers to the *w*-th high-frequency words. *Weight*_*w*_ stands for the weight of *w*. All weights are taken as 1, *TimesInTitle*_*w*_ denotes the number of times *w* appears in the title, and *imesInContent*_*w*_ refers to the number of times *w* appears in the text.

The longer the text, the higher the probability of containing high-frequency words, which would diminish the importance of shorter texts and so bias our analysis. To overcome, this problem, we, therefore, scaled the importance of the text with a length, specifically,


$$G9_{i}=TextImportance_{i}$$

$$=\sum_{w}w\times Weight_{w}\times TimesInTitle_{w}$$

$$+Sacle\left(\frac{\sum_{w}w\times Weight_{w}\times TimesInContent_{w}}{Length_{i}}\right)$$

where $Scale\left(W_{i}\right)=W_{i}\times \frac{TitleImportance_{max}}{W_{max}}\times 2$ is equivalent to mapping the distribution interval of the importance of the text to the distribution interval of two times the importance of the title, thereby reflecting the importance of the text.

(iii) Text clustering:

The Latent Dirichlet Allocation topic modeling method was used for cluster analysis. Then the k-means clustering method was used to iterate 20 times, and the clustering results and topic words of each category were calculated. The resulting text topic categories are shown in Table 4.

**TABLE 4:** Results of text clustering.

Category	Words
The Global COVID-19 Pandemic	China, Pandemic, Country, COVID-19, America, Rumor, Virus, Global, International, Nationwide, Time, Organization, Vaccine, World, Pneumonia, My country, Cooperation, Media, Netizen, Anti-pandemic
Resumption of Work and Production	Enterprise, Pandemic, Resume work, Service, Policy, Support, Resume production, Produce, Related, Prevention and control, Influence, Employment, Guarantee, Provide, Handle, Period, Unit, Department, Funds, Development
Economic construction	Development, Construction, Job, Advance, Xi Jinping, Innovation, Promote, City, Get rid of poverty, Economic, Meeting, Industry, Project, Accelerate, Center, Promote, Tack, Perfect, System, Governance
Transportation and Travel	Shanghai, Reservation, Time, Open, Restore, Traveler, Information, Citizen, Traffic, Tourist, Park, Travel, Tourism, Service, Vehicle, Scenic spot, Passenger, Culture, Railway, Health
Pandemic Prevention and Control	Pandemic, Prevention and control, Gob, Personnel, Beijing, Pneumonia, Do well, Health, Measure, COVID-19, Management, Community, Implement, Guarantee, Hubei, Situation, Isolation, News, service, Focus
Pandemic Information Release	Case, Confirmed, Patient, Hospital, Pneumonia, Wuhan, Add, Discharged, COVID-19, Grand total, Hubei, Enter, Abroad, Report, Treatment, Severe, detect, Isolation, cure, new
Market Supervision	Consumption, Market, Sale, Vegetable, Price, Distribution, Case, Weather, Agriculture, Supermarket, Wildlife, Legal, Commodity, Consumer, Food, Illegal, Party, Store, Product, Release
Anti-Pandemic Measures	Mask, Disinfect, Health, Protection, Contact, Wear, Suggest, Symptom, Infection, Ventilation, Place, Coronavirus, Prevention, Virus, Do well, Pneumonia, Beijing, Period, Disease, New
The Front Line of the Battle Against the Pandemic	Pandemic, Wuhan, Hospital, First line, Medical Team, Gob, Time, People, Anti-pandemic, Child, Player, Volunteer, Doctor, Medical Personnel, Life, Nationwide, Party member, Hubei, Hope, Fight
Community Pandemic Prevention	Wuhan, Community, Hubei, Residents, Supplies, Community, Pandemic, Personnel, Mask, Service, Gob, Staff member, Street, Company, Introduction, Group, Prevention and control, Volunteer, Employee
Resumption of Classes and Employment	School, Student, Start of school, Time, Education, College, Back to school, Gob, Examination, Enrollment, Sign up, Recruitment, Study, Graduate, Candidate, Profession, Post, Related, Middle school, Information

(iv) Sentiment text analysis:

We use the *Sogou Sentiment Dictionary* as the basis of word classification. We give sentiments scores to each sentence according to sentence patterns and the frequency of emotional words, adverbs, negative words, and so on. Then we take the average of all sentence scores as the sentiment score of the text. The sentiment scores of all the texts were then linearly mapped to the interval of [−100, 100] to obtain G10. Daily average sentiment scores (see Figure 2) are within the range of [−0.26, 16.04]. The sentiment tends to be neutral and positive, but fluctuations can be observed at some important time nodes, such as a significant drop for the national day of mourning on 4th April.

**FIGURE 2:**
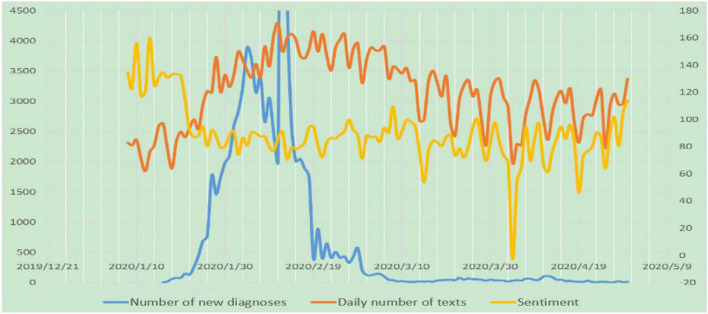
The trend of three sets of data.

### Data description and visual presentation

#### Data description

Of the 13,723 items of textual information incorporated within this study (see above), 9,735 (70.94%) elements were related to the pandemic. After 20 January, 12,765 items were posted, of which 9,705 (76.03%) concerned the pandemic. Here, we make preliminary observations on the collected and calculated data.

The data source for the number of newly confirmed cases was the official website of the National Health Commission of the PRC. For the period 16 January 2020 to 30 April 2020, we collected information on the daily number of newly confirmed cases in China (31 provinces, autonomous regions, and municipalities; and Xinjiang Production and Construction Corps). On 12 February, the 15,152 newly clinically confirmed cases (including 13,332 clinically confirmed cases in Hubei province) were recorded. However, to facilitate observation of the overall trends, we set the upper limit of the scale of the spindle axis at 4,500, thus data for 12 February could not be shown.

It can be observed from the graph that starting from 22 January, with the intensification of the outbreak in China and the closure of Wuhan on 23 January, the sentiment score of the texts published on the WeChat official account exhibited a substantive downward trend. On 4 April, the national day of mourning, the sentiment score was at its lowest negative. After 27 April, the sentiment score experienced an upward trend. The sentiment expressed in the texts of the WeChat official account was, therefore, closely related to the severity of the pandemic.

From 23 January, the number of daily texts on the WeChat official accounts began to increase. This trend continued until 14 March, when the quantity started to decline. A development that might be interpreted through success in starting to control the spread of COVID-19. Information released through government WeChat accounts was closely associated with the stage of the pandemic. Overall, the government succeeded in spreading information and emotional guidance during the critical period of the pandemic.

#### Data subject category analysis

Python performed a cluster analysis on the text of the title and body, and 11 categories were generated; specifically (C1) The Global COVID-19 Pandemic; (C2) Resumption of Work and Production; (C3) Economic Construction; (C4) Transportation and Travel; (C5) Pandemic Prevention and Control; (C6) Pandemic Information Release; (C7) Market Supervision; (C8) Anti-Pandemic Measures; (C9) The Front Line of the Battle Against the Pandemic; (C10) Community Pandemic Prevention; and (C11) Resumption of Classes and Employment (see Table 5).

**TABLE 5:** Distribution of text categories in different official accounts.

	The global COVID-19 pandemic	Resumption of work and production	Economic construction	Transportation and travel	Pandemic prevention and control	Pandemic information release	Market supervision	Anti-pandemic measures	The front line of the battle against the pandemic	Community pandemic prevention	Resumption of classes and employment	Total
China Government network	87	422	46	21	248	173	25	61	17	18	19	1137
Communist Party Member	60	50	128	13	118	122	15	36	376	29	5	952
Communist Youth League Central Committee	454	37	24	10	65	30	35	46	689	27	140	1557
Beijing Release	8	131	110	112	604	146	19	66	40	27	40	1303
Shanghai Release	35	242	166	637	255	314	236	209	131	50	224	2499
Hubei Release	20	131	168	54	773	462	75	71	154	355	24	2287
Wuhan Release	20	126	88	137	418	509	139	92	182	585	38	2334
Beijing Center for Disease Control and Prevention	5	1	3	6	76	124	2	228	15	0	8	468
Hubei Center for Disease Control and Prevention	5	0	1	1	12	13	0	197	11	0	1	241
Total	731	124	759	1027	2676	2173	608	1098	1745	1118	547	13723

There were interesting differences in the coverage and the topics published by the various government WeChat official accounts. As the official account of the central government, *China Government Network* focused more on C2, with emphasis on restarting the domestic economy. *Communist Party Member* and *Communist Youth League Central Committee*, as official CP accounts, focused more on C9, emphasizing progress in the frontline flight against COVID-19. The *Communist Youth League Central Committee* stressed information about the global pandemic (C1). Five local government official accounts – *Beijing Release, Shanghai Release, Guangdong Release, Hubei Release*, and *Wuhan Release* mainly considered C5 and C6, namely, prevention and control themes and information release. This selection reflected the functions of local government in controlling the spread of the outbreak and disseminating information about the pandemic in their locality. For such functional reasons, *Shanghai Release* paid particular attention to C4. Alternatively, at the epicenters of the Chinese outbreak, *Hubei Release* and *Wuhan Release* both stressed C10, specifically the role of the local community in pandemic prevention. Focusing on control and management of the pandemic and, of most importance, effective grassroots prevention activities. The local outbreak prevention departments also have their own verified WeChat official accounts, although the number following such accounts was low. As the WeChat official accounts, the *Beijing Center for Disease Control and Prevention* and the *Hubei Center for Disease Control and Prevention* stressed C8, reflecting the remit of those departments.

**FIGURE 3:**
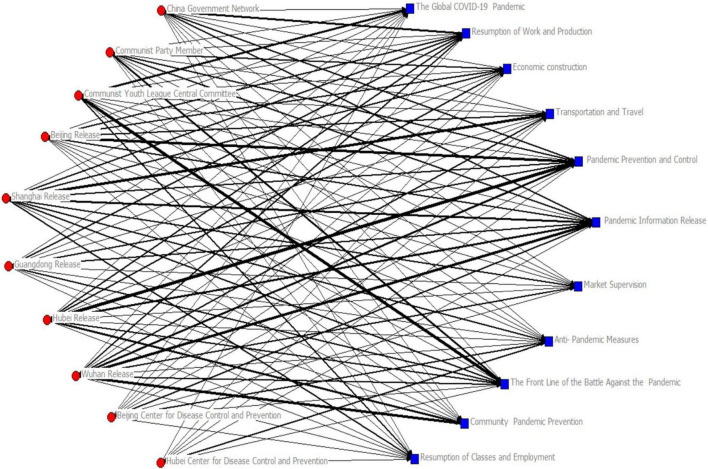
Distribution of text categories in different official account.

**FIGURE 4:**
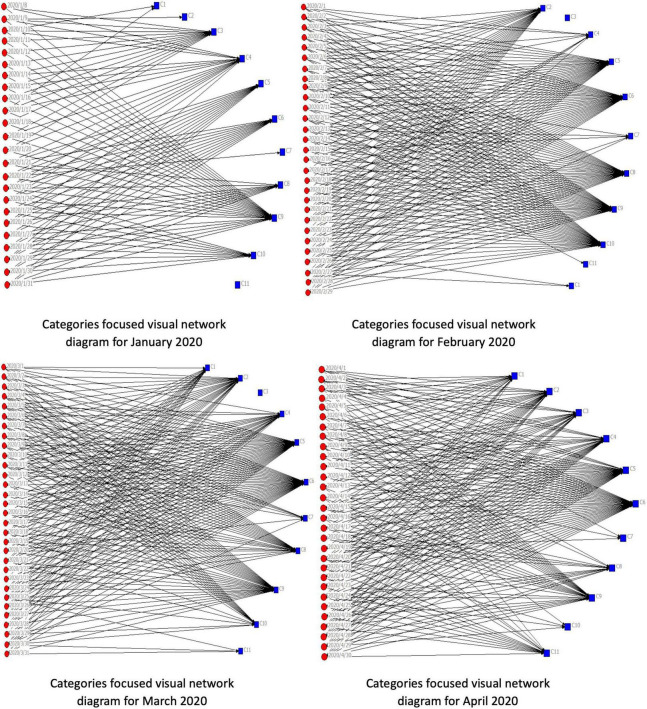
Categories focused visual network diagram for January 2021.

Next, a social network analysis of the matrix data was outlined. *Ucinet* software was deployed to analyze the network structure of the data matrix (see Figures 3, 4 and Table 5).

Based on the matrix data of “data-category,” the network diagram of two-mode is generated to analyze the focus of the government WeChat official accounts across our timeframe (see above).

During January, the 10 official accounts paid little attention to C1, C2, C7, or C11, which covered global, regulatory, and employment-production themes. Conversely, in January discussion about COVID-19 coalesced around themes such as C4 or C9. In particular, the arrival of the Spring Festival accounted for the emphasis on travel and transport matters, while reports about the initial spread of the virus in Wuhan explained the emphasis on C3 in the first half of the month, However, in the second half of January, with the outbreak in its early stages, the official accounts focused on themes such as C5, C6, C8, or C10, especially dissemination of information about COVID-19 and efforts to limit its spread.

It can be seen, from the network diagram for February, that the overall network density was significantly higher than in January. With the worsening of the severity of the outbreak, the focus on related themes intensified. The 10 official accounts barely focused on C3 or C11, both economic themes. At the end of the month, there was little attention paid toward global issues C1 or C4 or C7, transport, travel, and regulatory matters. This reflected the fact that China was at a crucial juncture in handling the medical emergency. Substantive parts of business and society were not functioning, and the state had issued instructions to prevent gatherings and reduce population movement. Throughout February, the emphasis was on C2 (resumption of work and production). C5 (pandemic prevention and control), C6 (pandemic information release), C8 (anti-pandemic measures), C9 (the front line of the battle against the pandemic), and C10 (community pandemic prevention). Most Chinese regions were at a high or the highest-level of public health emergency response. Government official accounts released a large amount of outbreak-related information on time, reflective (in part) of public hunger for information about the crisis. After the Spring Festival, and the peak period of the outbreak, the state aimed for an orderly resumption of work and production in different sectors, while fighting the pandemic.

As can be observed, from the network diagram, in March the 10 official accounts seldom focused on C3. At the start of the month, official accounts stressed C10 (community pandemic prevention), although this focus declined as control measures became more effective. From mid-to-late March, there was relatively little emphasis on C7 (market supervision). Meanwhile, the emphasis on transportation and travel (C4) increased as travel restrictions were liberalized. Simultaneously, the global spread of the pandemic was reflected in renewed stress on C1. At the end of March, increased attention was directed at C11 (resumption of classes and employment), reflective of expectations of the re-emergence of a more normal lifestyle. Throughout March, the pandemic remained an important topic on social media. There was an intensive focus on five themes – C2 (resumption of work and production); C5 (pandemic prevention and control), C6 (pandemic information release), C8 (anti-pandemic measures), and C9 (the front line of the battle against the pandemic). In March, China began to resume work and production in multiple industries, and, at the same time, the country was also in a critical period for pandemic preventive work.

In April, emphasis on C10 (community pandemic prevention) substantively diminished, reflecting the fact that the community prevention work was near its completion, although there was still a limited focus on C7 (market supervision). The restarting of the economy was reflected by a renewed emphasis on C3 (economic construction), in comparison with February and March. Official accounts also focused on C2 (resumption of work and production) and C11 (resumption of classes and employment). However, the emphasis on C8 (anti-pandemic measures) decreased significantly. This trend reflected two factors, first, the extensive media campaign meant that basic protective measures, such as increased ventilation, wearing masks, frequently washing hands, fewer social gatherings, and social distancing, had already been widely implemented. Second, evident progress made in containing the pandemic in China meant that some strict pandemic prevention measures were liberalized or cancelled. Furthermore, throughout April, the official accounts kept focusing on the five themes – C1, C4, C5, C6, and C9, reflecting, for instance, the global spread of COVID-19 and continuing attempts to control the pandemic.

## Data analysis and results

Drawing on Fox and Miler’s (1994) specification of warrants and guarantee conditions for discourse legitimacy, we developed six elements for both government and the public. First, for the government, we had Ga, Sincerity of interactive participation,” “Gb, Situation of interactive participation,” “Gc, Willing attention of interactive participation,” “Gd, Substantive contribution of interactive participation,” “Ge, Equal interaction,” and “Gf, Partial interaction.” Second, at the public level we specified “Pa, Sincerity of interactive participation,” “Pb, Situation of interactive participation,” “Pc, Willing attention of interactive participation,” “Pd, Substantive contribution of interactive participation,” “Pe, Equal interaction,” and “Pf, Partial interaction.”

The formulas for calculating the score of each element were as follows,


$$Ga_{Score}=\frac{G1+G2+G3}{3};Gb_{Score}=\frac{G4+G5+G6}{3};$$

Gc_Score=(G7+G8)/2;

$$Gd_{Score}=\frac{G9+G10}{2};Ge_{Score}=G11;Gf_{Score}=G12=\text{G}11/\text{P}4.$$

$$Pa_{Score}=P1;Pb_{Score}=P2;Pc\_Score=\left(P3+P4+P5\right)/3;$$

$$Pd_{Score}=P6;Pe_{Score}=P7;Pf_{Score}=P8=P4/P2.$$

First, the objective was to measure the *public energy field* of selected WeChat official accounts at both government and the public levels. Second, the *public energy field* was used to explore interactions between the various elements comprising the field. The government deployed its WeChat official accounts to publish and disseminate information and give the public a secure online interactive platform. Since the government controlled the screening, editing, and publishing of information, we assumed that the government-level *public energy fields* in the government WeChat public accounts were larger than the public-level energy fields. Of course, our study had been contextualized through the reality that, during the relatively severe stage of the pandemic outbreak, the degree of attention and interaction to incident information in cyberspace would be much greater than usual.

This study also assessed the interaction between government and the public. *Gf, Partial interaction* and *Pf, Partial interaction*, reflecting the *partial* public participation and the government’s response to this *partial* interaction. Since the values of these two elements were calculated through data from other indicators, they also reflected that *some people* were involved in the interaction. Gf and Pf were, therefore, excluded from our analysis to avoid multicollinearity when calculating the interaction of various elements.

### Social discourse between public and government

Summary statistics for Ga, Gb, Gc, Gd, Ge, and Pa, Pb, Pc, Pd, Pe were tabulated (see Table 6). This data indicated that Ga, Gb, Gc, Gd, Ge, and Pa, Pb, Pc, Pd, Pe were stationary. Results that allowed us to build a vector autoregression (VAR) model, and conduct likelihood ratio tests to detect whether interactions between government and the public were statistically significant. The model is based on the following formula:


(1) $$y_{t}=c+{\text{Φ}}_{1}y_{t-1}+{\text{Φ}}_{2}y_{t-2}+\cdots +{\text{Φ}}_{p}y_{t-p}+\varepsilon_{t},$$

**TABLE 6:** Descriptive statistics for Ga, Gb, Gc, Gd, Ge, and Pa, Pb, Pc, Pd, Pe.

	Minimum	Maximum	Average	Standard deviation	ADF test statistic	*P*-value
Ga	0	1	0.5006	0.1894	−11.2932	0.01
Gb	0	1	0.0883	0.1601	−22.1334	0.01
Gc	0	0.9854	0.3692	0.1522	−26.6127	0.01
Gd	0	0.7215	0.3454	0.0913	−17.9018	0.01
Ge	0	1	0.0077	0.0351	−22.8365	0.01
Pa	0	1	0.2707	0.3195	−28.2438	0.01
Pb	0	1	0.4327	0.4241	−28.9700	0.01
Pc	0	0.5994	0.0448	0.0764	−25.5811	0.01
Pd	0	1	0.0130	0.0356	−23.1734	0.01
Pe	0	1	0.0052	0.0401	−23.2137	0.01

where *y*_*t*_ refers to the 13, 728 × 10 matrix, and *p* stands for the order of VAR, which is selected based on Akaike information criterion (AIC). Here *p* = 3. The VAR model can be estimated by using the maximum likelihood (ML) method. If there was no significant interaction between the government and the public (*H*_0_), the coefficient matrices Φ_i_ would be block-diagonal, namely,


(2) $${\text{Φ}}_{\text{i}}=\left[\begin{array}{cc} {\text{Φ}}_{i,G} & 0\\ 0 & {\text{Φ}}_{i,P} \end{array}\right],$$

where Φ_i_ refers to the 10 × 10 matrix, and *0*’s stand for the 5 × 5 zero matrices. Based on the restriction, we could obtain the restricted likelihood (${ℒ}_{0}^{*}$). If there was social discourse between government and the public, all off-diagonal entries for Φ_i_ would be free to deviate from zero, and the corresponding likelihood function would be referenced as the unrestricted likelihood (${ℒ}_{1}^{*}$). In general, we expect ${ℒ}_{0}^{*}\leq {ℒ}_{1}^{*}$, because ${ℒ}_{0}^{*}$ is obtained under restriction. However, if *H*_*0*_ is valid, we would expect that ${ℒ}_{0}^{*}$ and ${ℒ}_{1}^{*}$ were close to each other. Based on this intuition, we formulated the likelihood ratio test based on ${ℒ}_{0}^{*}$ and ${ℒ}_{1}^{*}$, which, under the null, follows χ^2^ distribution with 150 degrees of freedom, therefore, $2\left({ℒ}_{1}^{*}-{ℒ}_{0}^{*}\right)$ = 1364.63. It can be seen that the statistic was highly significant, and *H*_*0*_ was rejected at even the 1% significance level. Thus, we could safely conclude that there was a significant social exchange between the public and the government.

We also considered the Granger causality test (Granger, 1969), which is a statistical concept of causality based on predictability. Here, we appraised the influence of the public and the government regarding our five dimensions (see the start of section “Social discourse between public and government”). Considering Ga and Pa, if the public’s sincerity of interactive participation did not affect that of the government, we would expect that the inclusion of Pa in the regression analysis would not improve the predictive power of a time series model based on Ga only. If the inclusion of Pa could improve the predictive power in a significant fashion, we might conclude that the sincerity of interactive participation from the public (Pa) influenced that of the government. These results are tabulated in Table 7.

**TABLE 7:** The Granger causality test between Ga, Gb, Gc, Gd, Ge, and Pa, Pb, Pc, Pd, Pe.

Null hypothesis	Test statistic	*P*-value	Null hypothesis	Test statistic	*P*-value
Ga Granger causes Pa	14.2549	0.00	Pa Granger causes Ga	10.1460	0.00
Gb Granger causes Pb	7.1564	0.00	Pb Granger causes Gb	6.7218	0.00
Gc Granger causes Pc	22.8300	0.00	Pc Granger causes Gc	8.0561	0.00
Gd Granger causes Pd	22.5712	0.00	Pd Granger causes Gd	8.9893	0.00
Ge Granger causes Pe	3.8649	0.01	Pe Granger causes Ge	0.6075	0.61

The government’s social discourse, in terms of sincerity, situation, willing attention, substantive contribution and equal interaction, had a significant influence on the public’s social discourse. However, equal interaction on the public side (Pe) did not Granger cause that of the government, or equivalently, it did not have predictive power over the *Ge, Equal interaction*.

**FIGURE 5:**
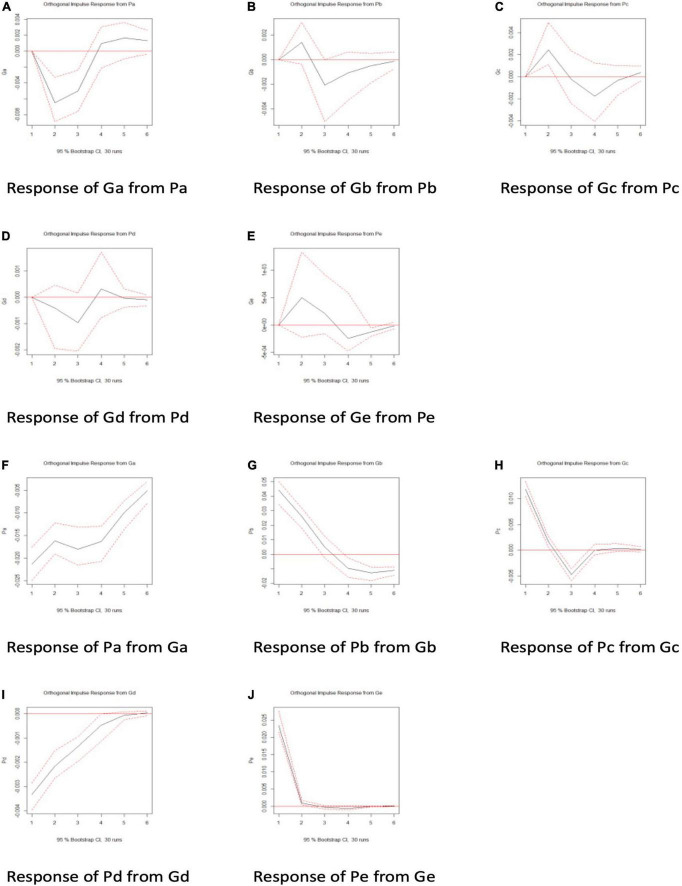
**(A–J)** The impulse response functions between Ga, Gb, Gc, Gd, Ge, and Pb, Pl, Pc, Pd, Pe.

The Granger causality tests are only beneficial for determining whether the influence was significant, not the direction of influence. To remedy this deficiency, we deployed the orthogonal impulse response function. The results are summarized in Figure 5 and the red lines indicate a 95% confidence interval.

In general, the plots from Figures 5A–E implied that the public first reacts negatively to “Ga, Sincerity of interactive participation” in a significant fashion; “Gb, Situation of interactive participation” had no significant influence on the public; “Gc, Willing attention of interactive participation” first enhanced public interaction significantly; “Gd, Substantive contribution of interactive participation,” and “Ge, Equal interaction” also had no significant effect initially on the public. Furthermore, the plots from Figures 5F–J suggested that Pa, Pb, Pc, Pd, and Pe lead immediately and significantly to the changes in Ga, Gb, Gc, Gd, Ge; and that it is worth noting that the situation of interaction, the willing attention of interaction and the equality of interaction were reduced in a significant fashion with the increase in those government indicators. To some extent, therefore, the government’s social discourse weakens that of the public. In general, the analysis based on the orthogonal impulse response function suggested that social discourse between the government and the public was unbalanced, with each of the five indicators implying that the government took the lead. The imbalance in dialog also reflected governmental control over the information released in WeChat official accounts. The amount of information posted by the public on those accounts was relatively small. However, it did not affect the equal dialog, because the government’s WeChat official account supplied the channel for interactive dialog.

### Social discourse analysis based on principal components

Principle Component Analysis (PCA) was undertaken on Ga, Gb, Gc, Gd, Ge, and Pa, Pb, Pc, Pd, Pe to further refine the analysis. We hypothesized that these principal components might explain 80% of variations in Ga, Gb, Gc, Gd, Ge and Pa, Pb, Pc, Pd, Pe, respectively. Three main components were extracted from the government dimension, namely, *Fac*1.*Government, Fac*2.*Government*,and *Fac*3.*Government*. Two main components were extracted from the public dimension, namely, *Fac*1.*Public* and *Fac*2.*Public*. Referring to the component score coefficient matrix, the calculation formula for each component score was obtained as follows:


Fac1.Government=0.142Ga+0.580Gb-0.562Gc-0.192Gd+0.044Ge

Fac2.Government=0.551Ga-0.016Gb+0.073Gc+0.679Gd-0.005Ge

Fac3.Government=0.080Ga+0.201Gb+0.181Gc-0.073Gd + 0.938Ge

Fac1.Public=0.445Pa+0.411Pb+0.197Pc+0.173Pd-0.220Pe

Fac2.Public=-0.249Pa-0.123Pb+0.289Pc+0.247Pd+0.802Pe

*Fac*1.*Government* was mainly composed of “Gb, Situation of interactive participation,” which reflected context and relevance of governmental social media interaction and its emphasis on the originality and design of information release. *Fac*2.*Government* primarily comprised “Ga, Sincerity of interactive participation” and “Gb, Situation of interactive participation” and reflected the government’s sincere and effective participation in social media interaction as well as generation of relevant content. *Fac*3.*Government* was largely comprised of “Ge, Equal interaction,” reflective of the equal dialog in social media, with the government paying attention and responding to public comments.

*Fac*1.*Public* overwhelming comprised “Pa, Sincerity of interactive participation” and “Pb, Situation of interactive participation.” This outcome reflects the public’s sincerity and the relevance of the public’s communication with the government on social media. If the public become active followers, they will pay attention to the information released by the government. *Fac*2.*Public* mainly reflected “Pe, Equal interaction,” which was derived from the social media dialog. The public notice governmental feedback on comments, while the content of texts released by the WeChat official accounts stimulated dialog and opinion exchanges between the parties. The five components of *Fac*1.*Government, Fac*2.*Government, Fac*3.*Government*, *Fac*1.*Public*, and *Fac*2.*Public* were used to assess each WeChat official account in each month and so evaluate the relevant *public energy field.* Results are shown in Table 8.

**TABLE 8:** Principal component scores.

	*Fac1.Government*	*Fac2.Government*	*Fac3.Government*	*Fac1.Public*	*Fac2.Public*
	Gb	Ga & Gd	Ge	Pa & Pb	Pe
Total	5534.964	6166.774	461.777	4498.014	585.154
China Government Network	666.847	543.126	32.298	466.779	29.121
Communist Party Member	481.526	407.876	17.602	345.221	37.458
Communist Youth League Central Committee	657.286	629.708	55.666	758.845	53.381
Beijing Release	675.181	661.314	34.02	329.785	53.95
Shanghai Release	710.815	955.673	160.413	931.244	151.586
Guangdong Release	474.71	434.807	18.146	309.246	38.273
Hubei Release	662.457	1080.404	61.103	571.104	92.571
Wuhan Release	709.472	1154.171	65.817	604.877	99.797
Beijing Center for Disease Control and Prevention	312.146	203.83	10.086	120.393	19.031
Hubei Center for Disease Control and Prevention	184.525	95.864	6.357	60.545	9.986
January	868.989	916.037	63.105	767.929	95.993
February	1740.512	2111.143	140.488	1405.243	185.391
March	1596.912	1790.629	149.149	1258.323	174.372
April	1328.552	1348.965	109.034	1066.519	129.397

**FIGURE 6:**
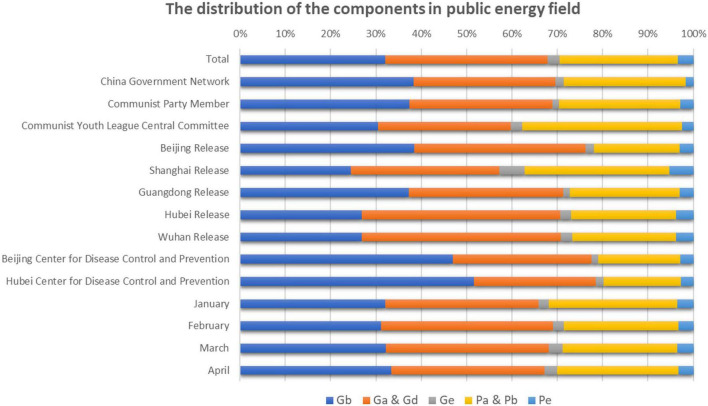
The distribution of the components in public energy field.

The scores obtained through using PCA were relative values. To further understand the data, we used an accumulation scale graph. The distribution of the diagram of the components was, therefore, constructed through those scores (see Figure 6).

Overall, the government’s *public energy field* was reflected through *Fac*1.*Government* and *Fac*2.*Government*, while the public’s *public energy field* was reflected in the *Fac*1.*Public*. However, the proportion of *Fac*3.*Government* and *Fac*2.*Public* were very low. Since the government controlled the release of information and the public was merely the recipient of information, the government ranked above the public in terms of discourse energy. The scores for *Fac*1.*Government* and *Fac*2.*Government* were, therefore, higher than those registered for *Fac*1.*Public*.

We observed and analyzed the data in Table 6. Comparatively, the number of followers for *Shanghai Release* was substantive and the public paid much attention to the information released by the municipal government. Overall, the public had a high degree of participation in interactive dialogs. The *Communist Youth League Central Committee* also had a large number of followers, and the public paid considerable attention to the information released. However, the public’s participation in interactive dialogs was slightly lower than for *Shanghai Release*. Because Wuhan and Hubei were seriously affected by COVID-19, the *public energy fields* for the government and the public registered for *Hubei Release* and *Wuhan Release* were higher than those of other local government WeChat official accounts. This finding implied that both the Wuhan government and the Hubei government were working hard to achieve timely release and disclosure of the information about the pandemic and that the public’s yearning for such information and interaction exceeded that of other localities. The *Beijing Center for Disease Control and Prevention* and the *Hubei Center for Disease Control and Prevention*, as well as the WeChat official account of the regional *Center for Disease Control and Prevention* (CDC), failed to register high scores for either *public energy field*, The low score for the public’s *public energy field* reflecting the modest number of followers, which was a consequence of public reading habits. The public concentrated on WeChat official accounts, as indicated by the large number of followers, of central, provincial, and municipal governments. The domestic pandemic information released through those accounts broadly satisfied public requirements, thus the public neglect of the CDC accounts. Given that the peak of the outbreak occurred in February and March, all *public energy fields* in February and March were higher than in January or April.

We also observed and analyzed the data in Figure 6. For the *Beijing Center for Disease Control and Prevention* and the *Hubei Center for Disease Control and Prevention*, the performance of *Fac*1.*Government* was outstanding. Those WeChat official accounts were performing effectively regarding “Gb, Situation of interactive participation.” The government’s inclination to participate in social media interaction was very strong – the proportion of *Fac*1.*Government* was significantly high.

For *Shanghai Release* and the *Communist Youth League Central Committee*, the performance of *Fac*1.*Public* was, again, outstanding. Those WeChat official accounts performed effectively concerning “Pa-Sincerity of interactive participation” and “Pb-Situation of interactive participation.” The number of followers and text readings on those WeChat official accounts was substantive, ensuring good public interaction. For *Wuhan Release* and *Hubei Release*, the performance of *Fac*2.*Government* was outstanding. Those two WeChat official accounts were effective for “Ga, Sincerity of interactive participation” and “Gd, Substantive contribution of interactive participation” As the epicenter of the epidemic, *Wuhan Release* and *Hubei Release* indicated that the government’s participation in such social media interaction was sincere and effective and that the government had made substantive efforts to edit content and release information about the disaster.

Next, we conducted a Granger causality test on the PCA results. The bivariate Granger causality test results are summarized in Table 9.

**TABLE 9:** The Granger causality test between Principle Component Analysis (PCA) results.

Null hypothesis	Test statistic	*P*-value	Null hypothesis	Test statistic	*P*-value
*Fac*1.*Government* **Granger causes** *Fac*1.*Public*	168.5707	0.00	*Fac*1.*Government* **Granger causes** *Fac2.Public*	27.50891	0.00
*Fac*2.*Government* **Granger causes** *Fac*1.*Public*	7.6425	0.00	*Fac*2.*Government* **Granger causes** *Fac*2.*Public*	22.5199	0.00
*Fac3.Government* **Granger causes** *Fac*1.*Public*	8.0188	0.00	*Fac3.Government* **Granger causes** *Fac*2.*Public*	6.1151	0.00
*Fac*1.*Public* **Granger causes** *Fac*1.*Government*	1.0125	0.39	*Fac*2.*Public* **Granger causes** *Fac*1.*Government*	4.6090	0.00
*Fac*1.*Public* **Granger causes** *Fac*2.*Government*	11.6780	0.00	*Fac*2.*Public* **Granger causes** *Fac*2.*Government*	41.9244	0.00
*Fac*1.*Public* **Granger causes** *Fac*3.*Government*	10.0071	0.00	*Fac*2.*Public* **Granger causes** *Fac*3.*Government*	15.7424	0.00

This analysis showed that the *Fac*1.*Public* did not Granger cause *Fac*1.*Government*. This finding confirmed the result of the orthogonal impulse response analysis, that government leads the social discourse process and the public follows. Moreover, given that the rest of the Granger causality tests are statistically significant, and that each of the PCA components comprised all five dimensions, these Granger causality results implied complex interactive relationships involving the government and the public.

We generated impulse response functions based on these principle components. The significance level was again set to 95%; and, for simplicity, we only incorporated significant results, when the confidence intervals did not contain zero during five periods.

**FIGURE 7:**
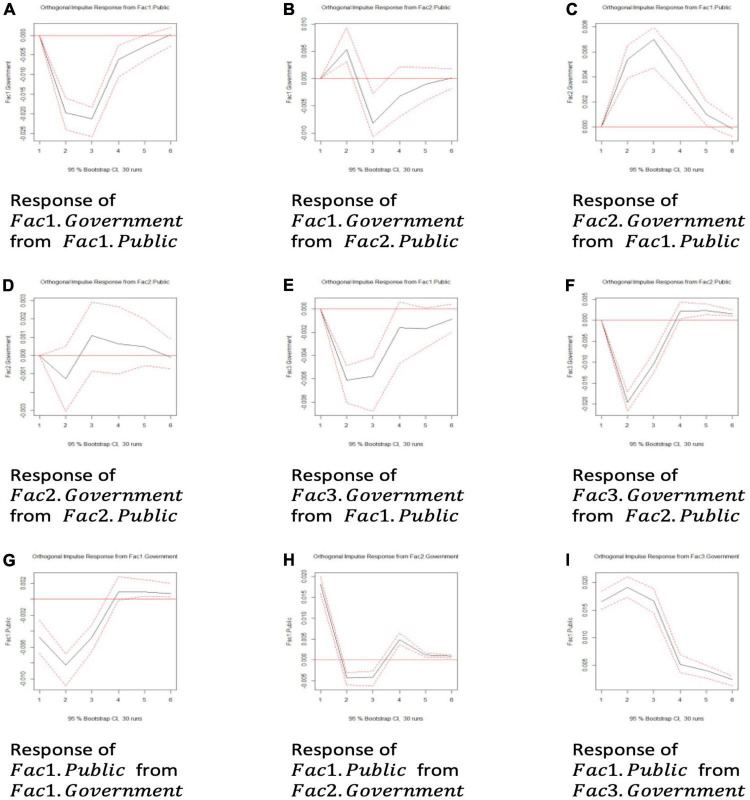
**(A–I)** The impulse response function between principle components.

As can be observed from Figure 7, all the impulse response functions were significant, apart from Plot (d). *Fac*2.*Government* reflected the government’s efforts in editing the released information; while *Fac*2.*Public* concerned the equality of social media dialog – whether WeChat official accounts had generated dialogs and genuine opinion exchanges between the government and the public. The insignificant result obtained from Plot (d) (see Figure 6) implied, however, that the interaction between the public and the government was unbalanced with the government prevailing and that the government should improve information release to balance the *public energy field*.

### Has China delayed dissemination of COVID-19-related information

Since the outbreak of the COVID-19 pandemic, the Chinese government has faced increasing criticism for its transparency in relation to the emergency. We, therefore, estimate whether the Chinese government delayed the release of vital information. We make the following three assumptions. First, it is not reasonable or even possible for China to withhold such details permanently; this information would be gradually disseminated to the public. The Chinese government might risk serious repercussions if the public eventually discovered that official information had been misleading. Second, delays in the release of information about COVID-19 might be interpreted through aims to prevent a public overreaction and allow the government to prepare its response. Third, these WeChat official accounts would be among the first sources to disseminate such information.

If these three assumptions held, we should find that some of Ga, Gb, Gc, Gd, and Ge; or alternatively, *Fac*1.*Government, Fac*2.*Government*, and *Fac*3.*Government* have predictive power over new COVID-19 cases. We establish two VAR models, the first of which comprised Ga, Gb, Gc, Gd, Ge, and new COVID-19 cases; while the second comprised *Fac*1.*Government, Fac*2.*Government, Fac*3.*Government*, and new COVID-19 cases; the Granger-causality test results are summarized in Table 10.

**TABLE 10:** Can government social discourse Granger cause the new diagnosis?

Null hypothesis	Test statistic	*P*-value
Ga Granger causes new diagnosis	125.3506	0.00
Gb Granger causes new diagnosis	125.3506	0.00
Gc Granger causes new diagnosis	7.949397	0.00
Gd Granger causes new diagnosis	22.68522	0.00
Ge Granger causes new diagnosis	30.53451	0.00
*Fac*1.*Government* Granger causes new diagnosis	0.7621662	0.52
*Fac*2.*Government* Granger causes new diagnosis	69.52285	0.00
*Fac*3.*Government* Granger causes new diagnosis	1.5368	0.20

These results implied that the government’s social discourse Granger causes the number of new diagnoses, namely, the government’s social discourse has predictive power over new diagnoses. This finding indicated that there might have been a time lag in releasing the information related to the COVID-19 pandemic. The Granger causality tests based on the principle components suggested that only *Fac*2.*Government* was statistically significant, whereas *Fac*1.*Government* and *Fac*3.*Government* were insignificant. Those results implied that the government dialogue lacked sincerity and contemporary relevance, which was consistent with the insignificant result of Plot (d) in Figure 6. The sincerity and situation of interactive participation from the government (*Fac*2.*Government*) were not affected by the public dialog (*Fac*2.*Public*).

**FIGURE 8:**
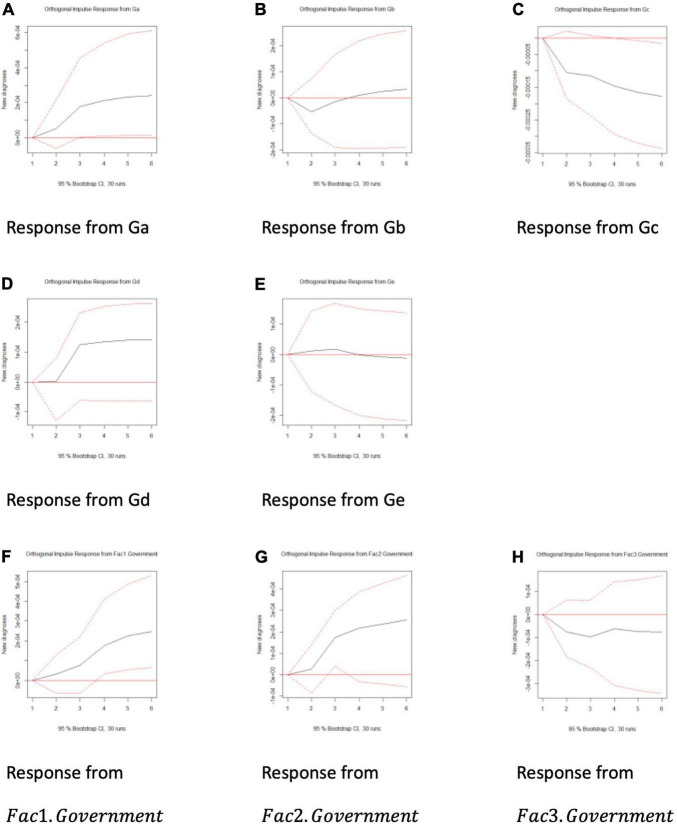
**(A–H)** The impulse response function of various factor on the number of new diagnosis.

If the government delays information dissemination, we would expect the relationship to be significant and positive. To explain the causal direction of this relationship, we generated orthogonal impulse response functions for various factors on the number of new diagnoses (see Figure 8).

The plots from (a) to (e) concerned the orthogonal impulse response functions of the number of new COVID-19 cases from Ga to Ge, while the plots from (f) to (h) were the impulse responses for three principle components: Fac1.Government, Fac2.Government, and Fac3.Government.

Arguably, if the government had delayed the dissemination of vital information, we should be able to observe significant and positive impulse response functions, indicating that an enhancement in social discourse on the government’s side increased new COVID-19 cases. From Plot (a) it can be observed that Ga has significant and positive impulse responses over a new diagnosis, implying potential information withholding. However, Plot (c) suggested that across a longer timeframe, the influence of Gc on new diagnoses was negative; therefore, perhaps the government’s interaction across a longer period might reduce the number of new COVID-19 cases.

Unfortunately, the first two principal components (Fac1.Government and Fac2.Government) suggested significant positive influences, with the third being negative, *albeit* it was insignificant. This finding implied that, perhaps, the negative influence in Plot (c) might be marginal, given that those three principal components explained 80% of the data variations.

Although this analysis implied that the government might have slowed the release of vital information about COVID-19, it did not exclude the possibility that the government might have supplied a reasonable prediction about the future course of the pandemic and, therefore, reacted proactively to combat the pandemic. The Chinese government might have adopted the predictions from the National Health Commission about future trajectories of the pandemic and moved in response to probable future trajectories. Of course, it might be explained through a time lag in CDC’s statistics on the new COVID-19 cases.

## Conclusion

Social media is being incorporated as an important governing tool (Bonsón et al., 2012; Mergel and Bretschneider, 2013), a process that, for instance, has occurred in China, principally through the establishment of official social media accounts. The importance of this new media has been seen through global trends to regulate social media communications (Al-Aufi et al., 2017) and growing governmental awareness of the importance of effectively engaging with public opinion through social media discourse. In particular, social media has the potential to increase governmental responsiveness to public attitudes and agendas (Al-Aufi et al., 2017). We have shown that the Chinese government and the public interact online to deal with major emergencies. Meanwhile, the establishment of proper discourse systems and *public energy fields* is required to create effective dialog interactions in social media, so that government and the public both benefit from these network platforms.

The conclusions generated through this study are as follows. First, analysis of (e) equal interaction and (f) partial interaction implied that the discourse system embodied through the government official accounts had legitimate conditions for *equal* and *some people* dialog. In such an equal space for dialog, both the government and the public can be energized and influence each other, conditions that assist the government in supplying public services and encourage the public to participate in shaping the public policy agenda. Nevertheless, our results show that the current dialog is unbalanced in favor of the government; circumstances derived from the fact that the public has relatively little information to disseminate. The government’s influence is, therefore, significant, as a consequence of its capacity to disseminate information through those accounts. Intensive public discussion of such details (of course) increases governmental influence regarding those nuggets of information, the converse public reaction diminishes such government influence.

Second, from the analysis of (a) sincerity of interactive participation, (b) situation of interactive participation, (c) willing attention of interactive participation, and (d) substantive contribution of interactive participation, we found that the government’s WeChat official accounts struggled to achieve good results in all four aspects simultaneously. Overall, our findings implied that the government should focus more on “situation of interactive participation,” “sincerity of interactive participation,” and “substantive contribution of interactive participation.” In response, the public should increase its emphasis on “sincerity of interactive participation” and “situation of interactive participation.” In summary, the government’s social discourse had a significant influence over the public’s social discourse, thus illustrating the potential of those WeChat official accounts as influential *public energy fields.*

Third, government official accounts with more followers had more energy and dialog interactions. Those WeChat official accounts, such as the *China Government Network, Communist Party Member*, the *Communist Youth League Central Committee*, the *Shanghai Release*, and the *Guangdong Release*, have many active followers and enhanced *public energy fields*. Overall, WeChat official accounts exhibited substantive virtual social capital (through many active followers), which facilitated information dissemination and government–public interaction. The accumulation of virtual social capital will intensify the influence of WeChat official accounts. Our study also suggested that WeChat official accounts should consider the influence of the followers, as well as the active interaction with the public and the continuous increase of the number of active followers, to increase their influence.

Fourth, in response to the COVID-19 outbreak in China, the government’s WeChat official accounts released comprehensive and timely information on 11 themes (see above). The number of posts, the emotional context of the texts, the topics of concern, and the number of readings were altered to reflect contemporary circumstances. It can be argued that the *public energy fields* presented by both the government and the public effectively portrayed and reflected the actual situation of the pandemic in China. However, the Granger causality tests revealed that the information content of the government accounts had predictive power over new COVID-19 cases, findings perhaps implying that there was a lack of transparency over the timeliness of the release of the information related to COVID-19. However, those results did not exclude the possibility that the government acted proactively in response to its predictions about the course of the pandemic. This outcome might also reflect the time lag in summarizing the new COVID-19 cases.

In general, those government WeChat accounts released information in a timely, comprehensive, and accurate manner, thereby meeting at least some of the public’s information requirements. Those accounts can guide and alter public behavior to assist with the resolution of social problems. Different WeChat official accounts have different *public energy fields* and concerns, reflecting contrasting organizational responsibilities and the accumulation of virtual social capital. Regarding the COVID-19 pandemic, although the *public energy field* of the government, reflected through the public accounts, was slightly larger than that of the public, the government had managed to generate substantive interaction with the public and assist the public in handling the pandemic. When the *public energy fields* of the government and the public approached equality, discourse power was more proportionate, and better communication and interaction were achieved. The government, therefore, has to further focus on the public’s social media requirements and enhance its promotion of active participation by the public. In summary, government WeChat accounts exist to provide information and conduct dialog with the public, thus facilitating wider participation in decision making. We need to translate online participation driven by social media into government behavior (Bertot et al., 2010).

## Data availability statement

The original contributions presented in this study are included in the article/Supplementary material, further inquiries can be directed to the corresponding author/s.

## Author contributions

CS and XG contributed to the ideas, manuscript architecture design, and manuscript writing. JS contributed to the application of statistical and mathematical to synthesize study data. MC performed the theoretical analysis. GL did the data crawling and cleaning. All authors contributed to the article and approved the submitted version.

## Footnotes

1
https://www.newrank.cn/
